# Trigger point dry needling increases masseter muscle oxygenation in patients with temporomandibular disorder*

**DOI:** 10.1590/1678-7757-2023-0099

**Published:** 2023-08-25

**Authors:** Carolina Ferreira de Macedo, Anelise Sonza, Alexia Nadine Puel, Adair Roberto dos Santos

**Affiliations:** 1 Universidade Federal de Santa Catarina Centro de Ciências Biológicas Departamento de Ciências Fisiológicas Santa Catarina Brasil Universidade Federal de Santa Catarina, Centro de Ciências Biológicas, Departamento de Ciências Fisiológicas, Programa de Pós-Graduação em Neurociências Florianópolis, Laboratório de Neurobiologia da Dor e Inflamação, Santa Catarina, Brasil.; 2 Universidade do Estado de Santa Catarina Programa de Pós-Graduação em Fisioterapia Santa Catarina Brasil Universidade do Estado de Santa Catarina (UDESC), Programa de Pós-Graduação em Fisioterapia, Florianópolis, Laboratório de Desenvolvimento e Controle Postural (LADESCOP), Santa Catarina, Brasil.; 3 Universidade do Estado de Santa Catarina Centro de Ciências da Saúde e Esporte Departamento de Fisioterapia Santa Catarina Brasil Universidade do Estado de Santa Catarina (UDESC), Centro de Ciências da Saúde e Esporte, Departamento de Fisioterapia, Programa de Pós-Graduação em Ciências do Movimento Humano, Santa Catarina, Brasil.

**Keywords:** Dry needling, NIRS, Muscle oxygenation, Masseter muscle, Craniomandibular disorder

## Abstract

**Background:**

Temporomandibular disorder (TMD) is an umbrella term encompassing various clinical complaints involving the temporomandibular joints, masticatory muscles, and/or associated orofacial structures. Myogenous TMDs are the most frequent cause of chronic orofacial pain. Musculoskeletal pain is commonly associated with myofascial trigger points (MTPs), for which dry needling (DN) is a routine treatment.

**Objective:**

To investigate muscle oxygenation and pain immediately after DN application on an MTP in the masseter muscle of patients with myogenous TMDs.

**Methodology:**

Masseter muscle oxygen tissue saturation indices (TSI%) were assessed by near-infrared spectroscopy (NIRS) pre- and post-interventions by a randomized, controlled, double-blind, crossover DN/Sham clinical trial (primary outcome). Pain was investigated by the visual analog scale (VAS). In total, 32 individuals aged from 18 to 37 years who were diagnosed with myogenous TMD and myofascial trigger points in their masseter muscles participated in this study. Relative deltas for the studied variables were calculated. Data normality was tested using the Shapiro-Wilk test. According to their distribution, data were analyzed by two-way ANOVA and the Student's t-, and Mann-Whitney tests. Statistical analyses were performed using Prism^®^ 5.0 (GraphPad, USA).

**Results:**

We found a significant difference (2,108% vs. 0,142%) between masseter muscle TSI% deltas after the DN and Sham interventions, respectively (n=24). We only evaluated women since men refused to follow NIRS procedures. Pain increased immediately after DN (n=32, 8 men), in comparison to Sham delta VAS.

**Conclusion:**

These findings show an increase in tissue oxygen saturation in the evaluated sample immediately after the DN intervention on the MTP of patients’ masseter muscle. Pain may have increased immediately after DN due to the needling procedure.

## Introduction

Temporomandibular disorder (TMD) is a collective term that includes several clinical complaints involving the temporomandibular joints (TMJs), masticatory muscles, or associated orofacial structures.^1^ Such conditions can cause pain in the pre-auricular region, TMJ, or masticatory muscles; limitations or deviations in mandibular movements; and TMJ noises during mandibular function. Moreover, pain during the function and palpation of masticatory muscles is a clinical factor associated with TMD.^2^

Myogenous TMD is a common musculoskeletal condition that results in pain and disability.^3^ In Brazil, 36.2% of an evaluated population had a painful TMD, 29.5% of which had myogenous disorders,^4^ such as myofascial pain.

Myofascial pain is the most common muscle pain disorder,^5^ frequently manifesting itself as regional pain and accompanied by increased muscle tension and decreased flexibility. The main characteristic of myofascial pain is the presence of a myofascial trigger point (MTP).^6^ MTPs are defined as a hyperirritable point associated with a hypersensitive nodule in a taut band of muscle fibers^7,8^ that is palpable during physical examination. This point is painful on compression and can give rise to referred pain and sensitivity, motor dysfunction, and autonomic phenomena.^7^

Local oxygen saturation in MTPs remains below 5% of normal levels.^9^ Maintaining adequate blood flow provides the oxygen needed for the aerobic production of adenosine triphosphate (ATP) and removes the by-products of metabolic processes in working muscles.^10^ Near infrared spectroscopy (NIRS) is a non-invasive methodology to measure relative or absolute global tissue oxygenation in muscles. Light in the near infrared (NIR) region (~700-900 nm) can penetrate several millimeters into biological tissues, whose main absorbing chromophores in skeletal muscle are hemoglobin, myoglobin, and cytochrome oxidase.^11^ Hemoglobin and myoglobin contain an iron core within each heme that varies its light absorption in the NIR range based on whether or not oxygen is bound to it.^12^

Skeletal muscle contraction depends on the energy generated by ATP.^13^ Conversely, sustained muscle contractions can compress capillaries and produce muscle ischemia.^14^ Thus, a lack of energy can compromise the recovery of Ca^2+^ by the sarcoplasmic reticulum due to the failure of the ATP-dependent calcium pump and result in the failed release of the actin-myosin complex (which also depends on ATP), perpetuating the cycle.^8^ The proposed *energy crisis theory* postulates that the sustained shortening of the sarcomere can compromise local oxygenation and nutrition and cause an energy crisis.^15^

This first theory was corroborated and expanded by the *motor endplate hypothesis*, which identifies dysfunction in the region of the extrafusal motor endplate, i.e., in the neuromuscular junction, as the main cause of MTP. A small amount of activity in the endplate causes some degree of muscle shortening.^16^ The acidic pH and calcitonin gene-related peptide (CGRP) in the active MTP^17^ favor increased acetylcholine (ACh) activity in the motor plate, leading to persistent muscle fiber contraction.^14^ These characteristics are found in all active MTP, including those in the masseter muscles of people with TMD. A study comparing healthy controls and adolescents with TMD showed a decrease of around 90% in masseter muscle oxyhemoglobin in a sample with myogenous disorders.^12^ This finding shows the importance of using technics to improve muscle oxygenation in individuals with myogenous TMD.

Dry needling (DN) the MTP in masticatory muscles is one of the treatments for patients with myogenous TMD that can decrease pain and dysfunction.^18,19^ Nevertheless, rigorous evidence on the physiological mechanisms and effects of DN is still needed for evidence-based level practice.^19^ Based on these premises, we consider the hypothesis that a DN intervention in masseter muscle MTPs of individuals with myogenous TMD would increase local tissue oxygenation.

## Methodology

### Ethics

The protocol of this study (CAAE: 88455018.9.0000.0121) was approved by the local Institutional Review Board at the Santa Catarina Federal University following the Declaration of Helsinki. Written informed consent was obtained from all participants and their legal guardians. This study is registered in the Brazilian Registry of Clinical Trials (ReBEC) under identification code RBR-7mwjzwb, accessible at https://ensaiosclinicos.gov.br/rg/RBR-7mwjzwb. This manuscript followed the CONSORT statement.

### Trial design

**Figure 1:**
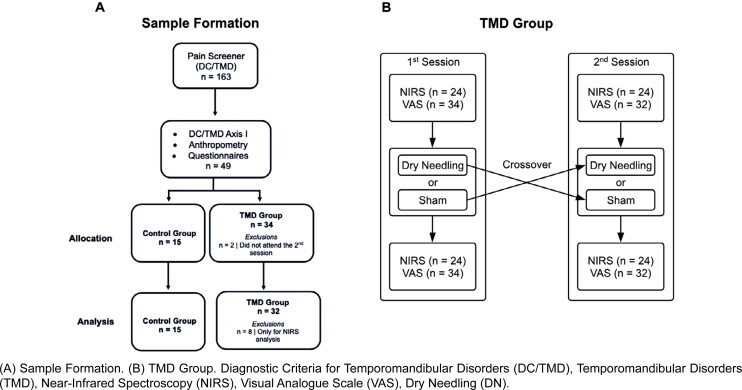
Protocol flowchart of this study

In total, two interventions were compared in this randomized, controlled, double-blind, crossover clinical trial. The flowchart and sample formation of this study are shown in Figure 1. The diagnosed TMD individuals who composed the experimental group — named TMD group (TMDG) — were randomized into two groups, the sham group (Sham) (with no active procedure) and the dry needling group (DG), in a cross-over design. Both interventions were applied on the same individual with a time interval of seven days between them (washout).^20^ The evaluator and patients were blinded to the type of applied intervention. Individuals without TMD composed the control-paired group (CG), which was only used to show the difference in TMD diagnosis and pain in comparison to the experimental group (which had the same anthropometric characteristics).

### Participants

Sample size was calculated based on data obtained in a pilot test with a mean ΔTSI% = 2 (primary outcome) and standard deviation=3. With an α=0.05 and β=0.20, the ideal sample size totals 24 participants, considering a design effect=1.25. Sample collection ended when the required number of individuals was reached for the main variable of interest (tissue oxygenation).

Myogenous TMD was diagnosed by a dentist who specialized in orofacial pain using the Diagnostic Criteria for Temporomandibular Disorders (DC/TMD)^3^ protocol.

To investigate the presence of MTP in patients’ masseter muscle, the following criteria were used during clinical palpations: the presence of a palpable taut band in the masseter muscle; the presence of a hypersensitive point within the taut band; and the replication of pain symptoms of patients with referred pain caused by trigger point.^9^

To be included in this study, participants also had to be at least 24 hours off any analgesics or anti-inflammatory drugs.^21^

Individuals aged under 18 years; pregnant women; people with vascular diseases, blood dyscrasias, trigeminal neuralgia, history of infectious odontogenic diseases in the previous 30 days, and/or history of cancer or radiotherapy in their head and neck regions; and those in the experimental group who failed to attend the two sessions were excluded from this study. Participants who refused to follow method procedures for specific variables were excluded from that specific analysis.

For sample selection, 163 university students were initially screened (Figure 1) using the DC/TMD Pain Screener.^3^ From these individuals, 34 were allocated to the TMDG and 15 to the CG. Based on inclusion criteria (TMD and MTP in the masseter muscle), individuals were invited to participate in this research and to sign an informed consent form. During this study, two individuals who participated in the first session and had their data collected missed the second session and were excluded from our sample. Moreover, all men (n=8) in the TMDG had to be excluded from NIRS data analysis as they refused to remove their beards, preventing the NIRS device from properly reading tissue oxygenation. The final sample for masseter muscle oxygenation included 24 women.

### Data collection procedures

Data were collected in a quiet room, whose access was restricted to researchers and study participants to ensure privacy.

Anthropometric assessments were carried out by measuring participants’ body mass with a digital scale (Orion^®^, Kikos, Brazil) and height with a stadiometer (Welmy, Brazil).

A Visual Analogue Scale (VAS) positioned horizontally was then shown to participants by a blinded evaluator, who asked them to mark their current level of pain on it. VAS is a validated (accurate) and reliable (reproducible) instrument to measure pain intensity for clinical and research purposes.^22^ In its scores, 0 represents no pain and 100, the greatest possible pain. VAS measurements were obtained from subjects of all genders with myogenous TMDs.

Immediately after the VAS measurement, the oxygenation of the masseter muscles on both sides of patients’ face was measured by the same blinded examiner using Near-Infrared Spectroscopy (NIRS) (Portamon, Artinis^®^, Netherlands).^23^ For this, participants remained seated with their knees and hips flexed at a 90° angle, feet resting on the floor, hands relaxed on their thighs, and their heads kept naturally in an upright position. The skin over the muscle was sanitized with 70% alcohol and the NIRS device was placed on the masseter-muscle belly and covered with a black cloth to prevent the passage of ambient light.^23^ The NIRS device was kept in position for two minutes to stabilize its reading before the measurement as indicated by the manufacturer. During the reading, subjects remained at rest for one minute. The same procedure was repeated on the opposite side. Trichotomy was necessary for individuals with hair in the masseter region since their beards prevented NIRS readings. Participants who refused to shave had to be excluded from the sample. This was a critical point in this study as it excluded all male participants from the experimental group from the NIRS assessment.

Then, the most sensitive trigger point of the masseter muscle was identified by palpation by the lead researcher, who marked its position on the skin with a white pencil and registered it on participants’ medical records.

The lead researcher used drawing envelopes to randomize the therapeutic intervention^24^ and, after skin asepsis with 70% alcohol, performed either deep dry needling or sham (inactive procedure) at the trigger point, as described below. Each enrolled participant and a secondary evaluator were blinded to the assigned procedure.^25^

Dry needling was performed with a sterile acupuncture needle (0.20 × 13 mm, Qinzhou, China) inserted into the most sensitive trigger point identified in the masseter muscle. After insertion into the skin with the aid of a cursor, the needle was deepened into the muscle and moved 10 times outward and inward without leaving the skin. Its direction was changed to various locations in the area at an average rate of one movement per second. Right after needle removal, light pressure was applied with a cotton pad at the puncture site to prevent bleeding. Then, the NIRS measurement was repeated by the secondary evaluator, (blinded to the administered therapy), enabling the verification of any change in tissue oxygenation. VAS measurements were also repeated by the blinded evaluator.

An acupuncture needle specifically adapted for this test was used in the sham procedure. A telescopic tube was fixed to a plastic base with 1 cm in diameter, which prevented the needle to penetrate the skin when manipulated, thus only simulating the sensation of the needle impact on the skin. This plastic base was immobile and fixed to the skin with a small piece of double-sided tape, being removed with a quick movement after one minute. Light pressure with a cotton pad was exerted on the site, as with the active DN procedure.

In the second session, after seven days of washout, participants were instructed to neither seek any type of intervention or treatment for TMD nor take any analgesics or anti-inflammatory drugs for at least 24 hours.^26^ NIRS measurements were repeated, completing the crossover between the DN and sham interventions (Figure 1). During the week between sessions, patients were instructed to avoid seeking any type of intervention or treatment for TMD, although the evaluator was available for help if needed.

### Statistical methods

Anthropometric, VAS, and muscle oxygenation data were tabulated in electronic spreadsheets (Excel^®^, Microsoft, USA). Then, relative deltas or overall change in the studied variables were calculated and statistical analyses were performed in Prism^®^ 5.0 (GraphPad, USA). Descriptive statistics were performed with mean and standard deviation values. Data normality was verified using the Shapiro-Wilk test. The Mann-Whitney test was applied for non-parametric data and the Student's *t*-test was applied for parametric data when appropriate. The level of significance was set at p<0.05.

Two-way ANOVA with repeated measurements was used to detect significant differences in the assessed variables. In the case of significant F values for ANOVA, the Tukey's post hoc critical difference test was used to identify the significant differences between means. The level of significance was set at p<0.05.

## Results

The experimental (TMDG) and control groups (CG) showed homogeneity regarding age and other anthropometric data (Table 1). However, groups differed in orofacial pain. Our final sample consisted of 32 individuals of all genders (F/M=88%/12%) aged from 18 to 37 years.

**Table 1:** Sample characterization regarding age, anthropometry, and orofacial pain

	CG		TMDG		
	Average	Standard Deviation	Average	Standard Deviation	*P*
Age (years)	21.00	3.10	21.53	3.79	0.763
Mass (kg)	67.44	15.75	60.87	10.66	0.240
Height (m)	1.68	0.11	1.66	0.06	0.322
BMI	23.58	3.71	22.12	3.33	0.178
VAS	0.00	0.00	20.25	17.92	<0.001
F/M (%)	73/27	-	89/11	-	-

Legend: Data were obtained from all participants and are expressed as means and standard deviations or percentages for the control group without TMD (CG, n = 15) and the group with TMD (TMDG, n = 32). Mass (kg); Height (m); Body Mass Index (BMI, kg/m2); Visual Analogue Scale (VAS); female (F); male (M).

**Figure 2:**
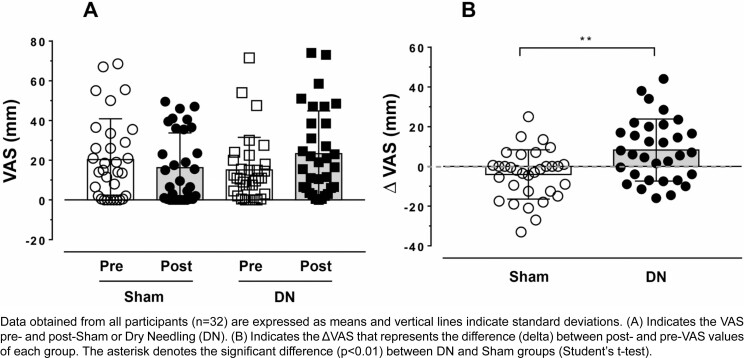
Pain intensity measured before and after DN or Sham using the Visual Analog Scale (VAS) of individuals with TMD

Figure 2 shows pain intensity according to the Visual Analog Scale (VAS). We observed a non-significant increase (F_(3, 93)_=2.647; p=0.054) between sham and DN pre- and post-procedures (Figure 2A) but found a significant difference (t_(df=31)_= 3.496; p=0.0015) between sham and DN ΔVAS in the ipsilateral masseter muscle (Figure 2B).

**Figure 3:**
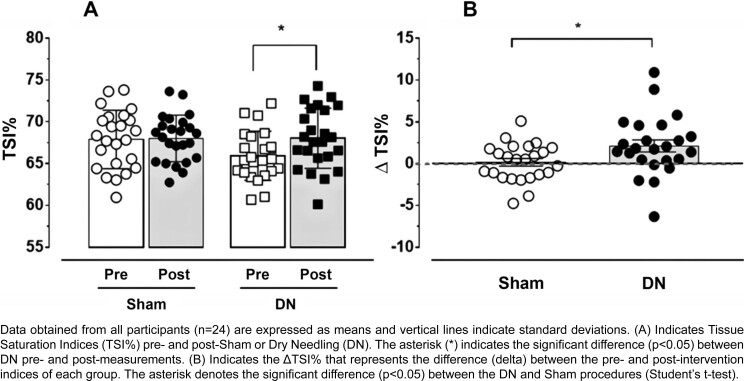
Tissue Oxygen Saturation Index (TSI%) of the masseter muscle of individuals with TMD

Figure 3 shows the tissue oxygen saturation indices (TSI%) of the masseter muscles that underwent intervention in the experimental group. Two-way ANOVA indicated a significant difference (F_(3, 69)_=3.623; p=0.017) between pre- and post-DN intervention assessments (Figure 3A). Furthermore, we found a significant difference (t_(df=23)_= 2.353; p=0.014) between variations in tissue saturation indices (ΔTSI%) in the DN and Sham interventions for the ipsilateral masseter muscle (Figure 3B).

Tissue oxygenation data in the masseter muscle contralateral to the Sham or DN interventions showed no significant difference (F_(3, 69)_=1.337; p=0.270) between pre- and post-intervention measurements. Additionally, we observed no difference (t_(df=23)_= 1.376; p=0.091) between the variations in tissue saturation indices (ΔTSI%) in the muscle contralateral to the intervention (contralateral data not shown in figures).

## Discussion

To the best of our knowledge, this is the first study that shows that DN significantly increases the tissue oxygen saturation (ΔTSI%) of the masseter muscle in individuals with TMD. This increase occurs ipsilateral — but not contralateral (without intervention) — to the intervention.

A previous study showed that DN sessions also increased tissue oxygen saturation in the trapezius muscle.^27^ For this, that research used laser Doppler and white light spectroscopy to measure the perfusion and oxygenation of the subcutaneous tissue to a depth of eight mm, respectively. Our results corroborate these findings and indicate that DN increases tissue oxygen saturation, despite the differing methods.

The DN technique uses needle insertion as in acupuncture. A study showed that acupuncture in the anterior region of the tibia of healthy women increased blood flow in the tibialis anterior muscle.^28^ The authors showed that the effect of acupuncture was directly proportional to needling intensity, i.e., the more intense the stimulation, the more pronounced the increase in blood flow. They observed such findings after superficially or deeply inserting a needle into the tibialis anterior muscle, which included manipulating the needle to provoke a distinct sensation of distension, heaviness, or numbness (*DeQi*). They used a customized non-invasive optical probe composed of six green and three near-infrared light-emitting diodes to measure blood flow in patients’ skin and muscle, respectively.^28^ They also found a similarly increased blood flow after a session in which they inserted a needle into the trapezius muscle and overlying skin of healthy individuals and of those with fibromyalgia and myalgia in the trapezius.^29^

The use of NIRS has also shown that acupuncture on the Achilles tendon of healthy men increased its blood volume and O_2_ saturation (StO_2_), which remained above pre-treatment levels for 30 minutes after needle removal.^30^ However, the number of studies evaluating the hemodynamic effects of acupuncture by NIRS is still considered limited.^31^

A mechanism that may explain the response of increased local muscle blood flow to needle stimulation is the release of vasoactive substances such as CGRP and substance P, activating Aδ and C fibers by the axon reflex and leading to vasodilation in small vessels and increased blood flow.^32^ In Wistar rats, CGRP essentially mediated antidromic vasodilation in skeletal muscles after electrical stimulation of the afferent C fibers (non-myelinated) in dorsal roots. This peptide may be important to clinically improve blood flow in skeletal muscles, such as that produced by acupuncture.^33^

However, some NIRS results showed no significant differences in total hemoglobin and StO_2_ in the trapezius muscle immediately after DN.^34^ The authors used six needles (0.2×40 mm) inserted around the NIRS sensor and left it in place for 15 minutes. Note that the authors ignored palpation of the trapezius muscle to find MTPs. They considered that pain points in the neck region are often close to the same anatomical point of the trapezius muscle, always positioned the NIRS sensor at that anatomical point.^34^

Note that as StO_2_ represents the balance between O_2_ supply and consumption, changes in StO_2_ reflect a flow change in the same direction and/or a metabolism change in the opposite direction. Therefore, proportional changes in flow and metabolism may be associated with an unchanged StO_2_.^35^

The discussion of our results should consider that, in the innervation of the masseter muscle, the proportion of myelinated (A) and unmyelinated (C) afferent fibers of the trigeminal nerve branches is greater than in the spinal nerves. Cranial nerves have relatively few type-C fibers, providing a higher conduction speed of trigeminal nociceptive signaling than spinal nerves and a shorter propagation distance to the head.^34^ Peripheral nerves in the head also have fewer sympathetic efferent axons than peripheral spinal nerves. Cutaneous blood vessels in the trigeminal region receive both sympathetic and parasympathetic innervation, unlike segmental levels, whose blood vessels receive no parasympathetic innervation.^36^ These differences and the anatomical-physiological peculiarities of the orofacial region may explain the different results across studies when compared to other parts of the body, including, for example, effects on the orofacial region to those on trunks or limbs.

Some individuals are genetically less susceptible to needle-induced nerve stimulation and are called weak responders. They have excessive amounts of endogenous opioid peptide antagonists or receptor deficiencies.^37^

Regarding the pain variable assessed using VAS, we observed a significant increase in the variation of pain intensity perception (ΔVAS) immediately after the DN procedure. The Sham intervention failed to result in any difference in our evaluation of this variable (Figure 3). This result shows that we performed the DN and Sham interventions correctly since Sham caused no pain. Some studies compared the acute effects of dry needling active the MTPs in the masseter muscle of patients with TMD, showing increased pressure pain thresholds immediately after the DN.^38^ The evaluation of these thresholds has a different action mechanism for measuring pain than VAS. Nonetheless, unlike our results, Bonora and Zugasti^39^ (2017) showed a significant decrease in VAS perception and sleep bruxism^39^ immediately after DN for TMD.

Pain after an MTP DN procedure is a common complication that usually lasts less than 72 hours and can occur due to damage to muscle fibers and intramuscular nerves, bleeding and transient inflammatory reactions. Therefore, method designs should consider pain after DN if they aim to investigate its therapeutic efficacy as this outcome can overlap with the original myofascial pain and influence patients’ classification.^40–42^

A review^43^ suggests a different treatment protocol from ours. It proposed at least six weekly sessions in which needles are retained for about 5 to 20 minutes at several sites near the likely source of nociception, achieving the *DeQi* sensation by initial and intermittent mechanical stimulation or administration of high-intensity sub-nociceptive electrostimulation. *DeQi* has been associated with sensations of nagging pain, heaviness, distension, numbness, tingling, heat, and spreading.

MTP needling therapies should be part of a broader treatment approach rather than being offered as a single intervention.^42^

Analyzing the sample characteristics in this study, most participants who had myogenous TMD were women. Epidemiological studies show the highest prevalence of pain in women.^44^ A systematic review has also shown that women are twice as likely than men to develop TMD.^45^ Thus, our results corroborate the relevance of gender in the development of TMD. Still, we should highlight that a limitation of our trial was that we only evaluated tissue oxygenation in women for the reasons described in the methods section. As a suggestion, future studies should address masseter oxygenation in men and women with TMD to understand if all genders show the same oxygen behavior and investigate different kinds of pain.

## Conclusion

DN significantly increased tissue oxygen saturation (ΔTSI%) in the masseter muscle of women with myogenous TMD. This increase occurred ipsilateral to the intervention but not contralateral (without intervention). Therefore, this study shows improved oxygen saturation in the masseter muscle due to DN in MTPs.

These data may provide useful additional knowledge for studies that analyze the therapeutic mechanism of this intervention when applied to the masseter muscle and its relation with pain types and origin.

## Data Availability

The datasets generated and/or analyzed in this study are available from the corresponding author upon reasonable request.
